# Relational needs frustration: an observational study on the role of negative (dis)engaging emotions

**DOI:** 10.3389/fpsyg.2023.1232125

**Published:** 2023-11-23

**Authors:** Davide Pirrone, Laura Sels, Lesley Verhofstadt

**Affiliations:** Department of Experimental Clinical and Health Psychology, Ghent University, Ghent, Belgium

**Keywords:** intimate relationships, emotions, relational needs, relationship conflict, observational study, video-mediated recall

## Abstract

The present study aimed to explore the role of partners’ negative engaging and disengaging emotions in dealing with the frustration of autonomy and relatedness needs during conflict. In an observational study, partners from 141 heterosexual couples participated in a conflict interaction task followed by a video-mediated recall procedure during which they reported their level of relational need frustration and their emotions experienced at different moments during the interaction. Results showed that in partners, more autonomy frustration, experienced at the beginning of the conflict, was accompanied by more concurrent negative disengaging emotions (anger, irritation), whereas more relatedness frustration was accompanied by more negative engaging emotions (hurt, sadness, disappointment). Additionally, the concurrent association between partners’ relatedness frustration and their experience of negative engaging emotions was negatively moderated by their own relatedness relationship beliefs (as assessed by background questionnaires), indicating that for individuals who considered relatedness to be less important, relatedness frustration and negative engaging emotions were more strongly linked than for people with high relatedness beliefs. Finally, negative engaging emotions – assessed at the beginning of the conflict – were associated with more relatedness frustration at a subsequent time point in the interaction in men, but not in women. This study contributes to our understanding of how partners’ negative emotions and the frustration of important relational needs are intertwined.

## 1 Introduction

When we think about past interactions within our intimate relationships, it is hard to judge our experience without also considering how we felt. Indeed, emotions reflect how well our relationships are going, whether we get what we need within our relationship, or whether our desires are frustrated (Bloch et al., 2014; Yoo et al., 2014; Overall et al., 2015; Vanhee et al., 2018).

Although there is theoretical consensus about the fact that emotions serve a social and goal-directed function within our intimate relationships (Hofer and Hagemeyer, 2018; Algoe et al., 2020), many questions remain unanswered. For instance, how does the frustration of important relational needs, such as the need for autonomy and relatedness seep through into a couple’s emotional life? Can emotions actually help partners to fix their frustrated relational needs? More specifically, do feelings of anger and irritation help to disengage from one’s partner and to achieve or restore a sense of independence in the relationship? Similarly, do feelings of sadness and hurt help to mutually engage relationship partners and increase their feeling of connectedness within the relationship? Given the lack of rigorous examination of these interesting and clinically relevant questions, the current study investigated the function of partners’ negative engaging and disengaging emotions in order to deal with the frustration of their need for autonomy and relatedness during conflict.

### 1.1 The socially (dis)engaging function of emotions

Most theoretical perspectives on emotions assert that emotions are primarily experienced, expressed, and regulated in response to other people, thus serving a *social function* (Parkinson and Manstead, 2015; Tamir, 2016; Keltner et al., 2019). In the existing literature, different social functions of emotions have been proposed. For instance, Barret and Nelson-Goen (1997) argue that emotions serve social regulatory functions such as signifying the importance of certain relationships and helping to maintain and restore these relationships when the need arises. In their literature review, Keltner and Kring (1998) listed informative, evocative, and incentive social functions of emotions. Gruenewald et al. (2007) suggested that emotions serve the function of protecting one’s self-evaluation from social threats. More recently, Keltner and Lerner (2010) identified emotions to have a social function at the individual, dyadic, group, and cultural level as they foster social interaction and social problem-solving.

Within the emotion domain, socially engaging and socially disengaging emotions constitute two different dimensions of emotions that map onto different poles of a so-called social engagement continuum of emotion (Kitayama and Markus, 1990; Markus and Kitayama, 1991b, 1994; Kitayama et al., 2000, 2006).

*Disengaging emotions* consist of emotions that increase the social distance between self and others (Boiger et al., 2022a,b). These emotions have also been defined as ego-focused (Markus and Kitayama, 1991a), autonomy-promoting (De Leersnyder et al., 2015), and distancing (Fischer and Manstead, 2008). Positive disengaging emotions, such as pride and feelings of superiority, highlight positive internal and self-defining attributes, thereby affirming the identity of the self as independent and disengaged from others (Vansteenkiste et al., 2020). Negative disengaging emotions (e.g., anger, irritation), that typically result from blocking one’s goals or needs, impose a threat to the sense of the self as an independent entity, motivate the person to eliminate this threat and to restore and assert the self’s independence (Fischer and Roseman, 2007; King et al., 2018; Roth et al., 2019). This motivational tendency toward independence affirms the sense of the self as an independent and interpersonally disengaged entity (Gillison et al., 2019; Vansteenkiste et al., 2020).

*Engaging emotions* consist of emotions that connect the self with others (Boiger et al., 2022a,b). These emotions have also been defined as other-focused (Markus and Kitayama, 1991a), relatedness-promoting (De Leersnyder et al., 2015), and affiliating (Fischer and Manstead, 2008). By experiencing positive engaging emotions (e.g., communal feelings, feelings of respect), people highlight their social interdependence, facilitating reciprocal well-intended behaviors that, in turn, provide a significant form of self-validation. Negative engaging emotions, such as sadness and being hurt, result most typically from one’s failure to participate fully in an ongoing relationship or to otherwise live up to the expectations of intimate others (Boiger and Mesquita, 2012; Rothman and Magee, 2016; Williams et al., 2018), therefore posing a threat to one’s sense of self as a fully interdependent entity (Kitayama et al., 2000). These emotions, in turn, motivate the person to eliminate the threat by restoring harmony or unity in the relationship and reaffirming one’s sense of self as an interdependent and interpersonally engaged entity (Roseman, 2011).

This distinction is also congruent with both clinical theory and research regarding the types of emotion that occur during conflicts in couples. For instance, an approach to couple’s therapy bearing substantial empirical support makes a difference between hard and soft emotions (Backer-Fulghum et al., 2018; Luginbuehl and Schoebi, 2020). Hard emotions are defined as emotions associated with asserting power and control that motivate people to protect themselves against partners who are perceived as harmful or neglectful, while soft emotions are pro-social emotions associated with experiencing or expressing vulnerability that lead to behaviors associated with closeness and relationship repair (Sanford, 2012).

Emotions play also a crucial role in interpersonal relationships, functioning not only as individual experiences but also as powerful communicative tools that shape interpersonal dynamics and establish recurring cycles of interaction (Butler and Randall, 2013). They serve as important signals and expressions of one’s internal states, needs, and intentions, conveying valuable information to others within the relational context (Barrett, 2017). Within intimate relationships, emotions create a ripple effect within interpersonal exchanges, influencing the emotional experiences and behaviors of both partners. When an individual expresses emotions, it can elicit corresponding emotional responses in their partner, initiating a reciprocal cycle of emotional exchanges that can either escalate or regulate the emotional climate between partners (Feeney and Fitzgerald, 2019). For instance, disengaging emotions communicate to the partner that the individual’s goals or needs are not met, serving as a cue for the partner to respect the expresser’s need for personal space and self-assertion (Boiger et al., 2022a,b). Contrarily, engaging emotions play a communicative role by signaling a desire for interpersonal closeness and serving as social signals to indicate the willingness to foster mutual and cooperative actions (Gilbert, 2022).

### 1.2 Goals and needs in intimate relationships

Given their high level of closeness, romantic partners have many opportunities to facilitate or obstruct each other’s goal pursuits within everyday interactions (Berli et al., 2018; Leung and Law, 2019; Brownhalls et al., 2021) and many relational need/goal theories have been proposed in the literature. For instance, the *Self-Expansion Model* highlights the centrality of relationship partners’ self-expansion and self-improvement goals in relationships (Aron et al., 2022). Additionally, relationship researchers have identified emotional involvement, companionship, security, intimacy, and sex, as essential relational goals in romantic relationships (Birnbaum and Reis, 2019; Brandão et al., 2020; Kluwer et al., 2020). Alongside these theories, it is important to acknowledge other therapeutic approaches that expand the understanding of relational needs. *Emotionally Focused Couple* therapists (EFT-C) consider the need for attachment, or one’s need for security and connection, as the most central need in intimate relationships (Johnson, 2004, 2009). Exploration and regulation of emotions are considered as a means to address underlying attachment and relational needs (Greenberg, 2004; Greenberg and Goldman, 2008). Within the relationship, partners should create an emotionally attuned and validating environment in which they can explore and address their psychological needs. Similarly, *Couples Schema Therapy* emphasizes the role of schema dynamics and underlying core psychological needs in shaping relationship patterns and interactions (Martin and Young, 2010). This approach recognizes that individuals bring core emotional and psychological needs, such as the need for love, safety, and validation, that can influence partners’ behavioral patterns within the couple dynamic. While each theory has unique characteristics, the focus on goals, needs, motivations, or values as central to the functioning of the romantic relationship is general.^1^

Within the broader psychological literature, one of the most prominent approaches to the conceptualization of basic psychological needs is *Self Determination Theory* (SDT; Ryan and Deci, 2022). According to SDT, individuals need to feel that their actions are self-directed and freely chosen (self-determined) rather than feeling forced by others, highlighting *autonomy* and *relatedness* as two fundamental psychological needs (besides the need for *competence*^2^) in people’s individual and relational well-being.

In romantic relationships specifically, the need for autonomy is defined as the need for a full personal endorsement of one’s own actions without feeling coerced or guilty toward the partner; a self-focused experience of volition and willingness within the couple (Deci and Ryan, 2014). Autonomy satisfaction in relationships results from partners being empathetic and supportive towards one another (Anderson, 2020). The need for relatedness in romantic relationships refers to the desire to form a meaningful relationship, care for the other, and to feel cared for by the other (Ryan and Deci, 2022). Relatedness satisfaction results from a genuine communication of care, interest, focus, and non-contingent support from one’s partner, and experiencing a successful stable bond with the partner in which one feels loved (Deci and Ryan, 2014; Knee et al., 2014).

Empirical evidence points at the importance of satisfying autonomy and relatedness needs in romantic relationships, for partners’ individual as well as relational well-being (Demir and Özdemir, 2010; Vandercammen et al., 2014; Wouters et al., 2014; Vanhee et al., 2016). However, while partners can be supportive towards each other relational needs, they can also frustrate their partners’ needs. SDT makes an explicit distinction between need satisfaction and *need frustration* in romantic relationships as they are regarded as separate concepts instead of opposites ends of a continuum (Bartholomew et al., 2011; Vansteenkiste and Ryan, 2013). Relational need frustration involves more actively and directly undermining a partner’s needs, as compared to more passively not satisfying one’s needs. As delineated by La Guardia and Patrick (2008), the frustration of relational needs occurs when partners feel controlled or pressured to behave in a certain way (autonomy frustration) or feel rejected and abandoned by their partner (relatedness frustration). In recent work, frustration of the need for autonomy and relatedness is documented to be associated with negative relationship outcomes (e.g., less relationship satisfaction, more conflict; see Vanhee et al., 2018).

### 1.3 Relational need frustration and emotions

In emotion science, *Appraisal theory* defines emotions as episodes in which the evaluation of an event in light of one’s needs – for instance, the evaluation of an event as frustrating one’s needs – leads to a cascade of changes (Scherer and Ellsworth, 2009; Moors et al., 2013; Moors, 2020). Thus, emotions act as *alarms* when people’s needs are incompatible or interfere with other people’s needs (Oatley and Johnson-Laird, 1987; Moors, 2007; Robinson, 2018; Sander et al., 2018).

In romantic relationships, this means that unmet or frustrated needs are expected to elicit specific emotions (Berscheid and Ammazzalorso, 2001). According to the SDT, negative emotions such as anxiety, grief, and anger are theorized to be typical responses to need frustration (Ryan and Deci, 2000). Indeed, there is some evidence that specific emotions result from partners’ needs being unmet or frustrated (Cupach et al., 2011; Diamond, 2014; Verhofstadt et al., 2020). Specifically, previous studies have documented the occurrence of sadness, anxiety, and anger when partners’ relational needs such as intimacy and belonging are unmet (Mikulincer and Shaver, 2007; Parrott, 2014). Direct empirical evidence for an association between relational need frustration and partners’ negative emotions (sadness, fear, and anger) was found in a recall study by Vanhee et al. (2018). Sadness was predicted by relatedness frustration in men and by autonomy frustration in women, whereas fear was only predicted by relatedness frustration in men. For anger, the results were comparable for men and women, with higher levels of autonomy frustration being associated with higher levels of anger.

In addition to their signal function, emotions have also a *communicative function*: they signal to the partner that needs are being frustrated within the relationship (Mazzuca et al., 2019; Benita et al., 2020; Cowen et al., 2021). In particular, it is theorized that emotions provide information about the expresser’s state, which can then result in different behaviors from the partner, such as being supportive and thereby reducing the expresser’s need frustration, or being affected by the expresser’s emotions in turn creating an escalation of frustration for both members of the couple (Van Kleef, 2009).

Previous studies indeed suggest that the expression of emotions varies in order to communicate specific needs. Disengaging emotions have been theorized to be expressed in the pursuit of ego-focused needs, while engaging emotions have been theorized to be expressed when individuals foster other-focused needs (Kitayama et al., 2006; Fischer and Manstead, 2008; Van Kleef et al., 2011). The implication might be that engaging and disengaging emotions differ from each other in terms of their underlying needs. Socially engaging emotions promote the achievement of what is best for the relationship with others (interdependent needs). Socially disengaging emotions foster the need of achieving what is best for an individual self (independent needs). However, to date, there is no empirical evidence to support these speculations, because studies on socially disengaging and socially engaging emotions have – to the best of our knowledge – never measured interdependent versus independent relational needs explicitly.

### 1.4 Romantic beliefs in intimate relationships

Existing literature showed that individuals enter romantic relationships with pre-existing beliefs about what those relationships should be like, which features make them satisfying or frustrating, and which relational needs should guide their behaviors as partners (Stackert and Bursik, 2003; Zagefka and Bahul, 2021). Such *relationship beliefs* make emotional responses to situations more fast as they suggest which cues are most important, the meaning of these stimuli, and the likely consequence of various courses of action (Baldwin, 1992; Crick and Dodge, 1994).

Partners’ responsiveness to each other’s relationship beliefs plays a crucial role in understanding emotional experiences during conflict. When partners fail to recognize or validate each other’s beliefs or the significance they attribute to particular relational needs, it can lead to a breakdown in mutual understanding and exacerbate emotional responses (Reis et al., 2004; Reis, 2012; Overall et al., 2015). For instance, if one partner highly values autonomy and seeks independence during conflict, but the partner fails to respect this need, it may intensify the emotions experienced, such as anger or resentment. Similarly, if one individual prioritizes relatedness, but the partner disregards or dismisses the importance of relatedness, it may heighten negative emotions, such as sadness or loneliness.

Relationship beliefs serve also as cognitive filters that shape how individuals perceive and interpret events within their relationship (Honeycutt and Cantrill, 2014). When individuals highly value a specific need, they tend to be more attentive and attuned to situations or behaviors that are relevant to that need. As a result, they may be more sensitive to detecting instances where the need is being threatened or unfulfilled, leading to heightened emotional responses when such frustration occurs (Vansteenkiste and Ryan, 2013). Furthermore, individuals who strongly believe in the importance of a particular need may have higher expectations for its satisfaction and may invest more effort in pursuing and maintaining it (Li and Fung, 2011). Consequently, when the need is frustrated, individuals might experience a greater sense of discrepancy between their desired state and the actual state of their relationship, leading to more intense emotional reactions.

It could thus be expected that relationship beliefs – how important for instance autonomy and relatedness are considered to be by relationship partners – may impact the partners’ emotional experience when these needs are unmet in their intimate relationship.

### 1.5 The present study

Despite the theoretical assumptions regarding emotions’ social function in the achievement of partners’ relational needs (Powers et al., 2011; Baker et al., 2014; Tracy, 2014), little is known about this association empirically. The available evidence for these arguments can be described as largely indirect and to our knowledge, a rigorous and interaction-based examination of the association between partners’ need frustration and their experience of (dis)engaging emotions is lacking from the current literature. Our study aims to contribute to the current literature by empirically exploring this association. We will do so during relationship conflict, a social context assumed to elicit relational need frustration, as it is defined as a situation in which partners interfere with each other’s needs (Bradbury et al., 2001; Whiting and Cravens, 2016).

We relied on a large sample of couples providing questionnaire data, and participating in a conflict interaction and video-mediated recall task, allowing us to assess both partners’ general as well as interaction-based level of autonomy and relatedness frustration, as well as the level of negative (dis)engaging emotions experienced during the interaction.

With regards to negative disengaging emotions, we expect that partners whose need for autonomy is frustrated during conflict, will experience more negative disengaging emotions. In turn, as negative disengaging emotions serve the social function of motivating people to eliminate threats to their need for autonomy and to restore and assert the self’s independence, we expect that partners’ negative disengaging emotions during conflict will lead to a decrease in their autonomy frustration. This means that partners’ reports of negative disengaging emotions during conflict will predict a decrease in their autonomy frustration at the next moment.

*H_1_*: Partners experiencing higher levels of autonomy frustration during conflict will report more negative disengaging emotions.

*H_2_*: Partners’ reports of negative disengaging emotions during conflict will predict a decrease in their autonomy frustration at the next moment.

With regards to negative engaging emotions, we expect that partners whose need for relatedness is frustrated will experience more negative engaging emotions. As these emotions motivate individuals to eliminate the threat to their need for relatedness by restoring the harmony and unity in the relationship, we expect that partners’ experience of negative engaging emotions will consequently lead to a decrease in their relatedness frustration.

*H_3_*: Partners experiencing higher levels of relatedness frustration during conflict will report more negative engaging emotions.

*H_4_*: Partners’ reports of negative engaging emotions during conflict will predict a decrease in their relatedness frustration the next moment.

It is important to acknowledge that the relationship between emotions and need frustration is bidirectional. Need frustration can lead to the experience of negative emotions, while the experience of negative (dis)engaging emotions can also impact the levels of need frustration. By exploring these bidirectional dynamics, we aim to contribute to the understanding of emotional regulation and conflict resolution processes within intimate relationships.

Finally, we predict that relational need frustration will more strongly predict negative (dis)engaging emotions when these needs are particularly important for people, meaning that they are aligned with their relationship beliefs. More specifically, we expect the experience of negative (dis)engaging emotions – resulting from partners’ relational need frustration – to vary as a function of their relationship beliefs, that is the importance partners assign to these needs in relationships in general.

*H_5_*: The association between partners’ autonomy frustration and negative disengaging emotions will be positively moderated by their own autonomy relationship beliefs.

*H_6_*: The association between partners’ relatedness frustration and negative engaging emotions will be positively moderated by their own relatedness relationship beliefs.

In order to test hypotheses 1 and 2 (and 5–6), we will examine partners’ concurrent levels of relational need frustration and emotions as experienced at a specific moment (i.e., near the beginning) in the conflict interaction. To test hypotheses 3 and 4, the cross-temporal association between partners’ emotions (experienced near the beginning of the conflict interaction) and relational need frustration experienced at a subsequent moment (i.e., near the end of the conflict interaction) in the interaction will be examined.

Emotional experiences often unfold in ways that highlight not only our own but also the partner’s involvement; as social interactions progress, we act and react to behaviors and feelings of our partners, as much as they react to our behaviors and feelings in turn (Butler, 2011). For this reason, the current study also aims to explore cross-partner effects. This means that we exploratively tested if people’s need frustration was associated with the emotions their partner experienced during the interaction.

## 2 Methods

The data used for this study was part of a larger national study, and has been used to investigate unrelated questions. Resulting publications can be found on osf.io/r732h. Materials used, relevant code, and data to conduct the reported analyses are available at https://osf.io/cuvj8.

### 2.1 Participants

A twofold recruitment strategy was used to collect data for this study: (1) a campaign was spread via posters in public places and via social media recruiting couples that were willing to participate in a research project on intimate relationships and (2) a team of research assistants recruited participants by means of a snowball-sampling technique. Couples that expressed interest in the study were informed about the project and evaluated for their eligibility to participate. To be eligible, couples had to be heterosexual, partners had to have been together for at least 1 year, and also living together for at least 6 months.

The final sample comprised 282 partners of 141 Belgian couples (aged 19–76 years, M = 36.34, SD = 13.93), with a range in relationship duration between 1 and 47 years (M = 12.91, SD = 11.99). More than half of the couples (51.1%) had at least one child, and 87.2% were married. In terms of educational level, the majority of the participants (42.9%) completed up to secondary school, 31.9% held a bachelor’s degree, 24.8% held a master’s degree, and 0.4% held a doctoral degree. The study procedures received positive advice from the Ethical Committee of the Faculty of Psychology and Educational Sciences of Ghent University.

### 2.2 Procedure

After providing their informed consent, participants were asked to independently complete an internet-based survey at home. To ensure the correct administration of the questionnaire, participants were provided with clear instructions and were encouraged to complete the survey independently at home. The survey allowed participants to respond to the items at their own pace. Additionally, participants were informed about the importance of providing accurate and honest responses to ensure the reliability and validity of the data collected. Thereafter, each couple was contacted in order to schedule an appointment in our lab for the observational part of the study. The laboratory session was composed of an 11-min videotaped conflict interaction task similar to the ones used in previous observational studies on couple conflict (Gottman and Levenson, 2002; Roberts et al., 2007), followed by a video-mediated recall task. At the end of this session, the couple took part in a debriefing with the responsible researcher and was compensated with 20 Euros for their participation in the study.

#### 2.2.1 Conflict interaction task

In the observational part of the study, the couples were asked to participate in a conflict discussion task that was similar to those used in previous laboratory studies on relationship conflict (Fletcher and Thomas, 2000; Simpson et al., 2003; Verhofstadt et al., 2005). The laboratory was set up as a living room and equipped to videotape the couples’ interactions. Before starting the interaction task, couples were asked to provide their written informed consent to be filmed. Next, both partners were separately asked to choose a salient relationship problem, from a provided list of conflict topics in romantic relationships, in which they had a desire for change. The topics (e.g., excessive demands or possessiveness, lack of equality in the relationship, frequent physical absence) were derived from previous work on sources of conflict within intimate relationships (Kurdek, 1994). After this topic selection had occurred, partners were randomly assigned to one of two conditions: *initiator* or *not initiator*. The conflict issue selected by the designated initiator was the one that the partners would discuss during their upcoming video-recorded interaction. The initiator was instructed to introduce the topic to the partner so that they could discuss this problem together. Both partners were instructed to discuss as much as they would do at home when experiencing a similar situation.

#### 2.2.2 Video-mediated recall task

At the end of the conflict interaction task, both partners separately completed a video-mediated recall task (Hinnekens et al., 2016). Partners viewed the video of their interaction on a laptop and were asked to re-experience the interaction. Every minute and a half, the video was automatically stopped (thus resulting in 7 stops) (Hinnekens and Kimpe, 2014), and partners were instructed to answer a range of questions about the interaction (e.g., write down the specific content of their thought at that specific point in time). Participants had the option to re-observe the last 10 s before the stop if they felt this would facilitate them to answer the questions.

### 2.3 Measures

#### 2.3.1 Interaction-based emotions

Interaction-based emotions were measured at the second stop (T_2_; after 3 min of interaction) and at the fifth stop (T_5_; after 7.5 min of interaction) during the video-mediated recall task. Using specific items from the Emotion Terms subscale of the CoreGRID instrument (Scherer et al., 2013), participants indicated the extent to which they felt irritated, angry, sad, disappointed, and hurt. Response options ranged from 1 = *completely untrue* to 7 = *completely true*. In line with previous literature (Markus and Kitayama, 1991b; Sanford and Rowatt, 2004), the following two scales were computed: (1) a *Negative Engaging Emotions* scale by averaging participants’ responses for the negative engaging emotion items (sad, disappointed, hurt; α_men_ = 0.76, α_women_ = 0.86), and (2) a *Negative Disengaging Emotions* scale by averaging participants’ responses for the negative disengaging emotion items (irritated, angry; α_men_ = 0.72, α_women_ = 0.82). Higher scores reflect higher levels of self-reported negative engaging and disengaging emotions, respectively.

#### 2.3.2 Interaction-based need frustration

At the second (T_2_) and fifth stop (T_5_) during the video-mediated recall task, participants were also asked to indicate the extent to which they at that specific time, experienced frustration of their need for autonomy (e.g., “At this moment, I was experiencing a lack of freedom of choice”) and relatedness (e.g., “At this moment, I was experiencing a lack of relatedness with my partner”) by means of a 7-point Likert-type scale (1 = *completely untrue* to 7 = *completely true*). Based on the SDT literature (Ryan and Deci, 2000; Deci and Ryan, 2014), each item was complemented with examples of each specific need frustration.

#### 2.3.3 Relationship beliefs

Participants’ beliefs regarding the importance of autonomy and relatedness in intimate relationships in general were assessed using two adapted items from the Need Satisfaction in Relationship Scale (La Guardia et al., 2000), which were included in the internet-based survey couples had completed at home. Using a 6-point scale (1 = *totally disagree* to 6 = *totally agree*) participants had to indicate their agreement with the following two statements: “In the best relationships, partners feel free to be who they are” and “In the best relationships, partners should feel connected to each other.”

#### 2.3.4 Global need frustration

Participants’ general levels of relational need frustration (autonomy, relatedness) were assessed using the Autonomy Frustration and Relatedness Frustration subscales of the Basic Psychological Need Satisfaction and Frustration Scale, adapted for use within intimate relationships (BPNSFS; Chen et al., 2015). The 8 items were scored on a 5-point Likert-type scale, ranging from 1 (*completely untrue*) to 5 (*completely true*). Each subscale consists of four items and measures respondents’ frustration of their need for autonomy (e.g., “In the relationship with my partner, I feel forced to do many things I would not choose to do”) and need for relatedness (e.g., “In the relationship with my partner, I feel that s/he is distant towards me”). Participants’ subscales scores were computed by averaging the responses for all items included in the specific subscale, with higher scores reflecting higher levels of need frustration. The internal consistencies for the autonomy and relatedness frustration scales were 0.75 and 0.71 for men, and 0.70 and 0.74 for women.

### 2.4 Data-analytic strategy

To investigate our research questions, we analyzed the data using multilevel Actor-Partner Interdependence Models (APIM; Kenny, 1996; Kenny et al., 2006). APIMs are used to study dyadic level data in which partners’ responses are non-independent. A person’s variable score is predicted by both his or her own predictor variable score (actor effect) and his or her partner’s predictor variable score (partner effect). Because we were working with partners that were distinguishable by gender, we first fitted models in which the effects of interest and variances could differ across gender, and compared these models with models for indistinguishable dyads (Kenny et al., 2006). Since the fit (as assessed by BIC/AIC^3^ values) improved significantly for the distinguishable models, we report the findings for these models.

**Figure 1:**
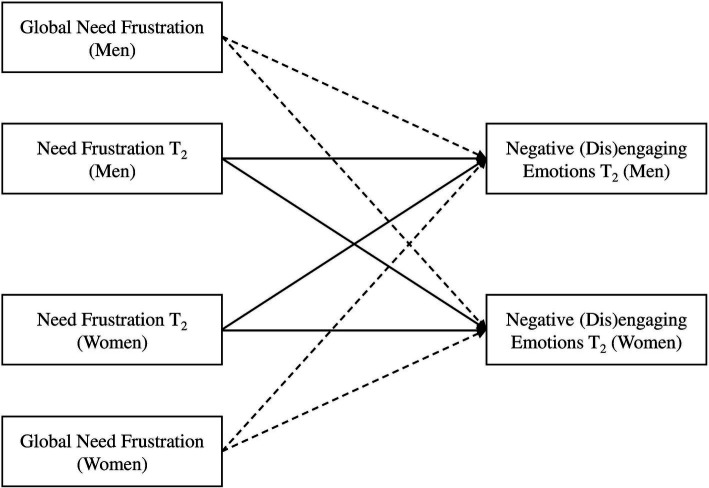
Actor-partner interdependence model used to assess the cross-concurrent associations between relational need frustration (autonomy, relatedness) at T_2_ and negative emotions (disengaging, engaging) at T_2_. The main paths are in black, while control paths are dashed.

First, we investigated the association between interaction-based need frustration (autonomy, relatedness) and participants’ concurrent experience of negative disengaging emotions (*H_1_*) and negative engaging emotions (*H_3_*). In model 1a, negative disengaging emotions at T_2_ were predicted by autonomy frustration at the same time point. In model 1b, negative engaging emotions at T_2_ were predicted by relatedness frustration at the same time point (Figure 1). We controlled for participants’ global level of autonomy and relatedness frustration to ensure that any observed effects were specifically attributed to the interactional needs frustration experienced during the conflict, rather than participants’ pre-existing global levels of frustration for these needs.

**Figure 2:**
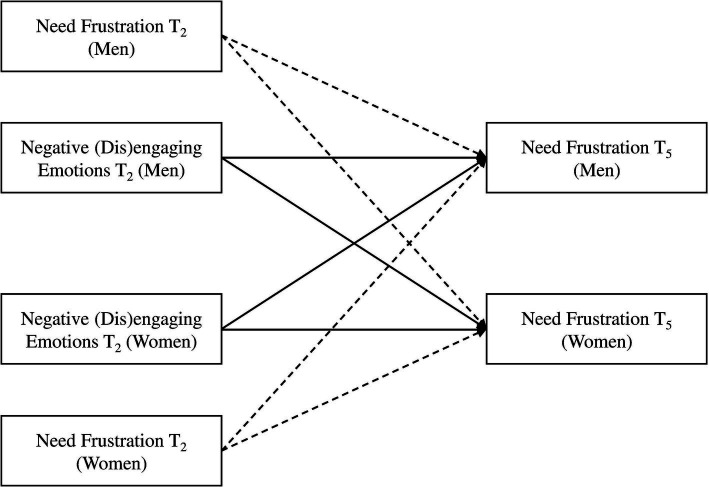
Actor-partner interdependence models used to assess the temporal associations between negative emotions (disengaging, engaging) at T_2_ and relational need frustration (autonomy, relatedness) at T_5_. The main paths are in black, while control paths are dashed.

Second, we investigated the effects of negative (dis)engaging emotions on participants’ subsequent autonomy frustration (*H_2_*) and relatedness frustration (*H_4_*). In model 2a, autonomy frustration at T_5_ was predicted by negative disengaging emotions at a previous time point (T_2_) controlling for autonomy frustration at T_2_. In model 2b, relatedness frustration at T_5_ was predicted by negative engaging emotions at a previous time point (T_2_), controlling for relatedness frustration at a previous time point (T_2_) to account for participant’s initial levels of relational needs frustration during the interaction, and examine the unique contribution of their emotions in predicting subsequent change in frustration of autonomy and relatedness (Figure 2).

**Figure 3:**
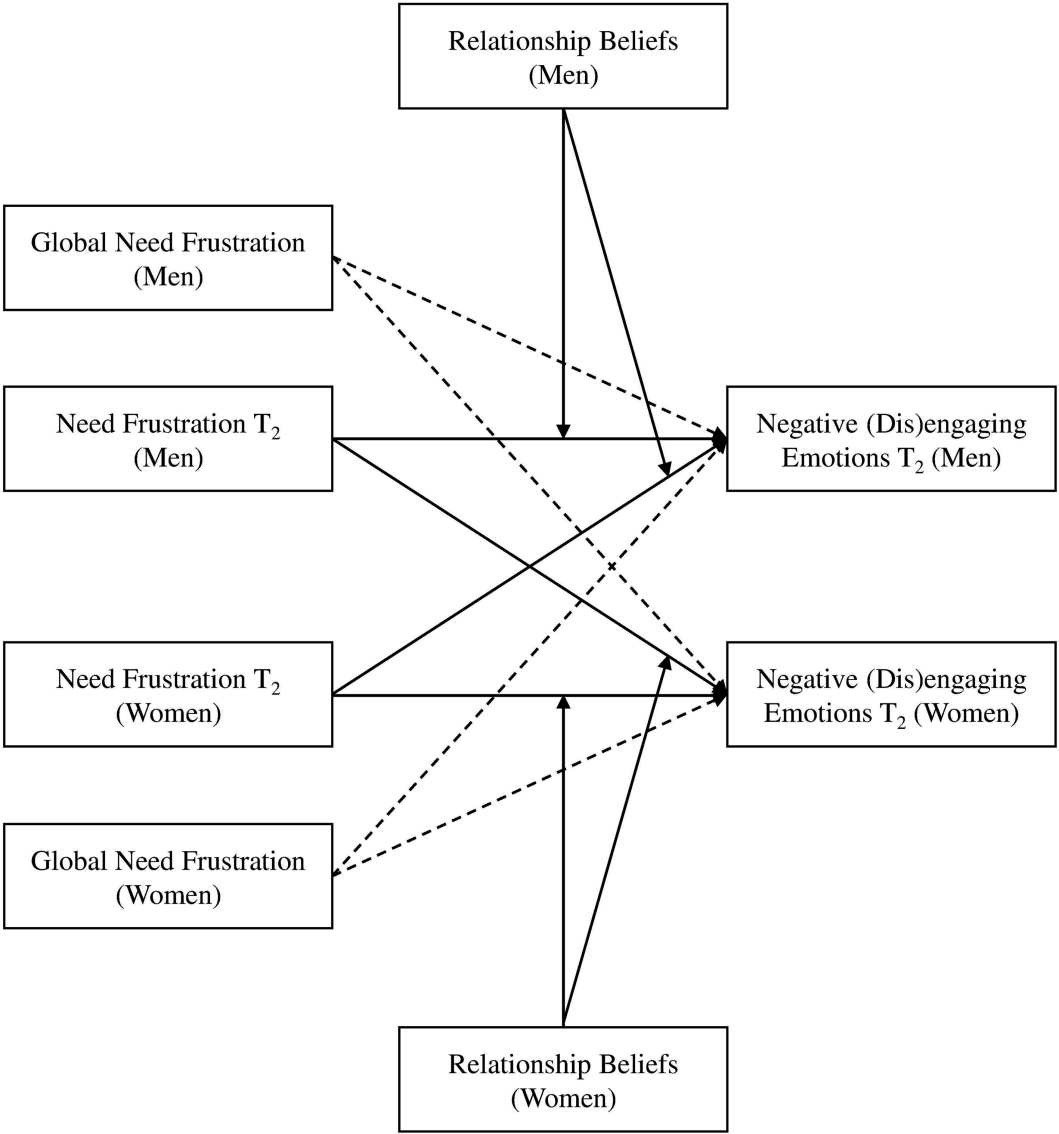
Moderated actor-partner interdependence models used to assess the cross-concurrent associations between relational need frustration (autonomy, relatedness) at T_2_ and negative emotions (disengaging, engaging) at T_2_. The main paths are in black, while control paths are dashed.

Third, we investigated the role of partners’ relationship beliefs in the association between relational need frustration and (dis)engaging emotions (*H_5_* and *H_6_*). In models 3a and 3b, we tested whether participants’ relationship beliefs (importance of autonomy and relatedness in intimate relationship) moderated the association between interaction-based autonomy and relatedness frustration (T_2_) on participants’ concurrent experience of negative disengaging emotions (*H_5_*) and negative engaging emotions (*H_6_*), respectively, controlling for participants’ global level of needs frustration (Figure 3). This control was not applied in models that investigated the relationship between emotional experience and subsequent interactional needs frustration (models 2a and 2b), because here we already explicitly captured change, by controlling for initial level of need frustration.

## 3 Results

Table 1 shows the descriptive statistics of the key variables, along with paired sample t-tests for possible gender differences in these variables, and Pearson correlation coefficients between all the key variables (Table 2).

**Table 1:** Descriptive statistics for key variables and results of paired sample t-tests comparing men and women.

	Men (*N* = 141)		Women (*N* = 141)		*t*	95% CI
	M	SD	M	SD		
Variables						
Global autonomy frustration	1.92	0.72	1.85	0.69	0.882	[−0.09; 0.24]
Global relatedness frustration	1.40	0.53	1.34	0.52	0.939	[−0.06; 0.18]
Autonomy relationship beliefs	5.19	0.74	5.35	0.69	−1.923	[−0.33; 0.00]
Relatedness relationship beliefs	5.31	0.71	5.43	0.66	−1.481	[−0.28; 0.04]
Autonomy frustration T_2_	2.43	1.60	2.10	1.49	1.850	[−0.02; 0.70]
Relatedness frustration T_2_	1.92	1.38	1.91	1.47	0.083	[−0.32; 0.35]
Negative disengaging emotions T_2_	1.94	1.21	2.20	1.54	−1.587	[−0.59; 0.06]
Negative engaging emotions T_2_	1.99	1.20	2.33	1.54	−2.064^*^	[−0.67; −0.02]
Autonomy frustration T_5_	2.38	1.52	2.14	1.53	1.288	[−0.12; 0.59]
Relatedness frustration T_5_	1.99	1.40	2.03	1.59	−0.198	[−0.39; 0.32]

^*^p < 0.05.

**Table 2:** Correlations between key variables.

		1	2	3	4	5	6	7	8	9	10
Principal variables	1. Autonomy frustration T_2_	0.379^**^	0.595^**^	0.367^**^	0.446^**^	0.553^**^	0.407^**^	0.128	−0.077	0.110	0.200^*^
	2. Relatedness frustration T_2_	0.418^**^	0.200^**^	0.332^**^	0.458^**^	0.433^**^	0.707^**^	−0.024	−0.018	0.200^*^	0.310^**^
	3. Negative disengaging emotions T_2_	0.525^**^	0.338^**^	0.242^**^	0.683^**^	0.270^**^	0.182^*^	−0.050	−0.092	0.285^**^	0.244^**^
	4. Negative engaging emotions T_2_	0.463^**^	0.454^**^	0.625^**^	0.404^**^	0.441^**^	0.410^**^	038	0.040	0.274^**^	0.280^**^
	5. Autonomy frustration T_5_	0.539^**^	0.417^**^	0.300^**^	0.263^**^	0.271^**^	0.492^**^	0.020	−0.097	0.050	0.198^*^
	6. Relatedness frustration T_5_	0.238^**^	0.621^**^	0.258^**^	0.471^**^	0.350^**^	0.048	−0.016	−0.087	0.165	0.390^**^
	7. Autonomy relationship beliefs	−0.059	−0.154	−0.070	−0.174^*^	−0.039	−0.116	0.274^**^	0.211^*^	−0.216^**^	−0.284^**^
	8. Relatedness relationship beliefs	−0.139	−0.202^*^	−0.163	−0.319^**^	−0.030	−0.228^**^	0.419^**^	0.076	−0.107	−0.092
Control variables	9. Global autonomy frustration	0.252^**^	0.243^**^	0.185^*^	0.199^*^	0.351^**^	0.132	−0.298^**^	−0.340^**^	0.244^**^	0.430^**^
	10. Global relatedness frustration	0.072	0.182^*^	0.108	0.216^*^	0.095	0.083	−0.417^**^	−0.363^**^	0.528^**^	0.220^**^

Correlations for women are presented above the diagonal, while correlations for men are presented below the diagonal. Correlations between men and women are presented on the diagonal. ^*^p < 0.05; ^**^p < 0.01.

Since the two scales for negative engaging emotions and negative disengaging emotion were highly correlated (*r*_m_ = 0.63; *r*_w_ = 0.68), we verified possible collinearity through the analysis of the Variance Inflation factor (VIF), which showed no significant collinearity between these two (with a VIF = 1.79, following the guidelines that a VIF > 4 indicates reasons for concern, and a VIF > 10 indicates serious multicollinearity; Brauner and Shacham, 1998; Belsley et al., 2005).

### 3.1 Model 1a: autonomy frustration (T_2_) on negative disengaging emotions (T_2_)

Results showed significant associations between autonomy frustration at T_2_ and negative disengaging emotions at T_2_ (actor effect), for both men and women, controlling for participants’ global level of autonomy frustration (Table 3). In line with our hypothesis (*H_1_*), participants who experienced higher levels of autonomy frustration during conflict interactions, also reported more concurrent negative disengaging emotions. None of the partner effect between autonomy frustration (global, interaction-based) and negative disengaging emotions at T_2_ were statistically significant.

**Table 3:** Results for the APIMs predicting negative disengaging and engaging emotions (T_2_) from men’s and women’s autonomy and relatedness frustration (T_2_), controlling for global relational need frustration.

	Estimate	SE	*p*	95% CI
Model 1a parameters				
*Intercepts*				
Men	1.88	0.09	0.000	[1.71; 2.06]
Women	2.26	0.12	0.000	[2.02; 2.49]
*Actor effects*				
Autonomy frustration_mT2_ → Disengaging emotions_mT2_	0.39	0.06	0.000	[0.27; 0.51]
Autonomy frustration_wT2_ → Disengaging emotions_wT2_	0.33	0.09	0.000	[0.16; 0.50]
Global autonomy frustration_m_ → Disengaging emotions_mT2_	0.09	0.13	0.762	[−0.22; 0.29]
Global autonomy frustration_w_ → Disengaging emotions_wT2_	0.48	0.18	0.007	[0.13; 0.83]
*Partner effects*				
Autonomy frustration_mT2_ → Disengaging emotions_wT2_	0.03	0.06	0.657	[−0.10; 0.15]
Autonomy frustration_wT2_ → Disengaging emotions_mT2_	0.03	0.08	0.729	[−0.13; 0.19]
Global autonomy frustration_m_ → Disengaging emotions_wT2_	0.21	0.13	0.114	[−0.05; 0.47]
Global autonomy frustration_w_ → Disengaging emotions_mT2_	0.30	0.17	0.084	[−0.04; 0.64]
Model 1b parameters				
*Intercepts*				
Men	1.98	0.09	0.000	[1.81; 2.16]
Women	2.35	0.12	0.000	[2.12; 2.57]
*Actor effects*				
Relatedness frustration_mT2_ → Engaging emotions_mT2_	0.36	0.07	0.000	[0.22; 0.49]
Relatedness frustration_wT2_ → Engaging emotions_wT2_	0.41	0.08	0.000	[0.25; 0.58]
Global relatedness frustration_m_ → Engaging emotions_mT2_	0.29	0.18	0.104	[−0.06; 0.63]
Global relatedness frustration_w_ → Engaging emotions_wT2_	0.40	0.24	0.097	[−0.07; 0.88]
*Partner effects*				
Relatedness frustration_mT2_ → Engaging emotions_wT2_	0.06	0.07	0.377	[−0.07; 0.19]
Relatedness frustration_wT2_ → Engaging emotions_mT2_	0.12	0.09	0.158	[−0.05; 0.30]
Global relatedness frustration_m_ → Engaging emotions_wT2_	0.08	0.19	0.689	[−0.30; 0.45]
Global relatedness frustration_w_ → Engaging emotions_mT2_	0.04	0.23	0.872	[−0.41; 0.48]

### 3.2 Model 1b: relatedness frustration (T_2_) on negative engaging emotions (T_2_)

Results indicated that the association between relatedness frustration at T_2_ and negative engaging emotions at T_2_ (actor effect), controlling for participants’ global relatedness frustration, was statistically significant for both men and women (Table 3). This was in line with our hypothesis (*H_3_*). People who experienced higher levels of relatedness frustration during conflict interactions, also reported more concurrent negative engaging emotions. There were no partner effects between interaction-based relatedness frustration and negative engaging emotions at T_2_.

### 3.3 Model 2a: negative disengaging emotions (T_2_) on autonomy frustration (T_5_)

Results disconfirmed our hypothesis (*H_2_*) that negative disengaging emotions at T_2_ would predict a decrease in autonomy frustration at a successive time point (T_5_), controlling for autonomy frustration at T_2_, as no effects were found for men or women (Table 4). Moreover, none of the partner effects of negative disengaging emotions (T_2_) on autonomy frustration at a later time point (T_5_) were significant.

**Table 4:** Results for the APIMs predicting autonomy and relatedness frustration (T_5_) from men’s and women’s negative disengaging and engaging emotions (T_2_), controlling for autonomy and relatedness frustration at previous time during the interaction (T_2_).

	Estimate	SE	*p*	95% CI
Model 2a parameters				
*Intercepts*				
Men	2.35	0.11	0.000	[2.13; 2.58]
Women	2.22	0.11	0.000	[1.99; 2.44]
*Actor effects*				
Disengaging emotions_mT2_ → Autonomy frustration_mT5_	0.04	0.11	0.672	[−0.16; 0.25]
Disengaging emotions_wT2_ → Autonomy frustration_wT5_	0.07	0.08	0.376	[−0.08; 0.21]
Autonomy frustration_mT2_ → Autonomy frustration_mT5_	0.45	0.08	0.000	[0.28; 0.61]
Autonomy frustration_wT2_ → Autonomy frustration_wT5_	0.51	0.08	0.000	[0.34; 0.67]
*Partner effects*				
Disengaging emotions_mT2_ → Autonomy frustration_wT5_	−0.12	0.08	0.120	[−0.27; 0.03]
Disengaging emotions_wT2_ → Autonomy frustration_mT5_	0.05	0.11	0.674	[−0.17; 0.26]
Autonomy frustration_mT2_ → Autonomy frustration_wT5_	0.20	0.08	0.018	[0.03; 0.36]
Autonomy frustration_wT2_ → Autonomy frustration_mT5_	0.06	0.08	0.500	[−0.11; 0.22]
Model 2b parameters				
*Intercepts*				
Men	2.05	0.09	0.000	[1.97; 2.32]
Women	2.00	0.10	0.000	[1.81; 2.20]
*Actor effects*				
Engaging emotions_mT2_ → Relatedness frustration_mT5_	0.32	0.09	0.001	[0.14; 0.50]
Engaging emotions_wT2_ → Relatedness frustration_wT5_	0.13	0.07	0.093	[−0.02; 0.27]
Relatedness frustration_mT2_ → Relatedness frustration_mT5_	0.54	0.07	0.000	[0.39; 0.68]
Relatedness frustration_wT2_ → Relatedness frustration_wT5_	0.71	0.07	0.000	[0.56; 0.85]
*Partner effects*				
Engaging emotions_mT2_ → Relatedness frustration_wT5_	−0.05	0.07	0.454	[−0.19; 0.09]
Engaging emotions_wT2_ → Relatedness frustration_mT5_	−0.06	0.10	0.566	[−0.24; 0.13]
Relatedness frustration_mT2_ → Relatedness frustration_wT5_	−0.08	0.07	0.242	[−0.22; 0.06]
Relatedness frustration_wT2_ → Relatedness frustration_mT5_	0.02	0.08	0.751	[−0.13; 0.18]

Due to the high correlation between negative disengaging emotions and negative engaging ones, we performed follow-up analyses, controlling for negative engaging emotions at T_2_ alongside autonomy frustration at T_2_. These analyses revealed similar results (Supplementary Table S1).

### 3.4 Model 2b: negative engaging emotions (T_2_) on relatedness frustration (T_5_)

In contrast to our hypothesis (*H_4_*) that negative engaging emotions at T_2_ would predict a decrease in relatedness frustration in the next moment, results showed that the actor effects of negative engaging emotion at T_2_ on relatedness frustration at a successive time point (T_5_) was statistically significant only for men, but in the opposite direction of what was expected. Men who reported more negative engaging emotions at the beginning of the interaction reported higher levels of relatedness frustration later on in the interaction. For women, no effect was found. Moreover, none of the partner effects of negative engaging emotions (T_2_) on relatedness frustration at a later point (T_5_), were found to be significant.

Again, we performed follow-up analyses, controlling for negative disengaging emotions at T_2_ alongside relatedness frustration at T_2,_ to look at the unique effect of negative engaging emotions (T_2_) on relatedness frustration (T_5_). Results indicated that the actor effects of negative engaging emotion at T_2_ on relatedness frustration at a successive time point (T_5_) were now significant for men and women (Supplementary Table S2). Specifically, higher levels of negative engaging emotions at T_2_, were predictive of more relatedness frustration at T_5_. Again, there were no partner effects.

### 3.5 Model 3a and 3b: moderating role of relationship beliefs

Lastly, we tested whether relationship beliefs about autonomy and relatedness moderated the actor and partner effects of autonomy frustration (T_2_) and relatedness frustration (T_2_) on negative disengaging (T_2_) and engaging emotions (T_2_), respectively (models 3a and 3b) by including relationship beliefs as main and interaction effects. Results of these analyses are presented in Table 5.

**Table 5:** Results for the moderated APIMs predicting negative disengaging and engaging emotions (T_2_) from the men’s and women’s autonomy and relatedness frustration (T_2_), controlling for global relational need frustration.

	Estimate	SE	*p*	95% CI
Model 3a parameters				
*Intercepts*				
Men	1.89	0.09	0.000	[1.71; 2.07]
Women	2.28	0.13	0.000	[2.03; 2.52]
**Main effects**				
*Actor effects*				
Autonomy frustration_mT2_ → Disengaging emotions_mT2_	0.39	0.06	0.000	[0.26; 0.51]
Autonomy frustration_wT2_ → Disengaging emotions_wT2_	0.36	0.09	0.000	[0.17; 0.55]
Autonomy beliefs_m_ → Disengaging emotions_mT2_	−0.02	0.13	0.870	[−0.28; 0.24]
Autonomy beliefs_w_ → Disengaging emotions_wT2_	−0.07	0.19	0.729	[−0.45; 0.31]
Global autonomy frustration_m_ → Disengaging emotions_mT2_	0.05	0.13	0.707	[−0.21; 0.32]
Global autonomy frustration_w_ → Disengaging emotions_wT2_	0.47	0.18	0.011	[0.11; 0.83]
*Partner effects*				
Autonomy frustration_mT2_ → Disengaging emotions_wT2_	0.03	0.09	0.637	[−0.14; 0.19]
Autonomy frustration_wT2_ → Disengaging emotions_mT2_	0.03	0.06	0.730	[−0.10; 0.16]
Global autonomy frustration_m_ → Disengaging emotions_wT2_	0.21	0.14	0.127	[−0.06; 0.48]
Global autonomy frustration_w_ → Disengaging emotions_mT2_	0.28	0.18	0.123	[−0.08; 0.63]
**Interaction effects**				
*Actor effects*				
Autonomy beliefs_m_*autonomy frustration_mT2_ → Disengaging emotions_mT2_	0.09	0.08	0.316	[−0.08; 0.25]
Autonomy beliefs_w_*autonomy frustration_wT2_ → Disengaging emotions_wT2_	−0.09	0.15	0.551	[−0.39; 0.21]
*Partner effects*				
Autonomy beliefs_m_*autonomy frustration_mT2_ → Disengaging emotions_wT2_	−0.04	0.08	0.657	[−0.21; 0.13]
Autonomy beliefs_w_*autonomy frustration_wT2_ → Disengaging emotions_mT2_	0.01	0.11	0.942	[−0.23; 0.24]
Model 3b parameters				
*Intercepts*				
Men	1.94	0.09	0.000	[1.77; 2.11]
Women	2.34	0.12	0.000	[2.11; 2.57]
**Main effects**				
*Actor effects*				
Relatedness frustration_mT2_ → Engaging emotions_mT2_	0.30	0.07	0.000	[0.17; 0.43]
Relatedness frustration_wT2_ → Engaging emotions_wT2_	0.45	0.09	0.000	[0.28; 0.62]
Relatedness beliefs_m_ → Engaging emotions_mT2_	−0.24	0.13	0.069	[−0.49; 0.02]
Relatedness beliefs_w_ → Engaging emotions_wT2_	0.08	0.17	0.647	[−0.26; 0.42]
Global relatedness frustration_m_ → Engaging emotions_mT2_	0.17	0.18	0.334	[−0.18; 0.52]
Global relatedness frustration_w_ → Engaging emotions_wT2_	0.37	0.24	0.127	[−0.11; 0.85]
*Partner effects*				
Relatedness frustration_mT2_ → Engaging emotions_wT2_	0.13	0.09	0.134	[−0.04; 0.30]
Relatedness frustration_wT2_ → Engaging emotions_mT2_	0.08	0.06	0.187	[−0.04; 0.21]
Global relatedness frustration_m_ → Engaging emotions_wT2_	0.07	0.18	0.713	[−0.30; 0.42]
Global relatedness frustration_w_ → Engaging emotions_mT2_	0.03	0.23	0.898	[−0.42; 0.48]
**Interaction effects**				
*Actor effects*				
Relatedness beliefs_m_*relatedness frustration_mT2_ → Engaging emotions_mT2_	−0.19	0.08	0.015	[−0.34; −0.04]
Relatedness beliefs_w_*relatedness frustration_wT2_ → Engaging emotions_wT2_	−0.24	0.12	0.038	[−0.47; −0.01]
*Partner effects*				
Relatedness beliefs_m_*relatedness frustration_mT2_ → Engaging emotions_wT2_	0.08	0.08	0.337	[−0.08; 0.24]
Relatedness beliefs_w_*relatedness frustration_wT2_ → Engaging emotions_mT2_	0.05	0.11	0.676	[−0.17; 0.26]

In contrast to our hypothesis (*H_5_*), results indicated that autonomy relationship beliefs did not moderate the association between autonomy frustration (T_2_) and negative disengaging emotions (T_2_) for both genders. Similarly, autonomy relationship beliefs were not a significant moderator of the partner effects neither for men, nor women.

Results disconfirmed our hypothesis (*H_6_*) that the association between participants’ relatedness frustration and negative engaging emotions would be positively moderated by their own relatedness relationship beliefs. Surprisingly, we found a negative moderating effect of relatedness relationship beliefs, indicating that relatedness frustration and negative engaging emotions were more strongly linked for individuals who considered relatedness to be less important than for people with high relatedness beliefs. This was the case for both men and women.

**Figure 4:**
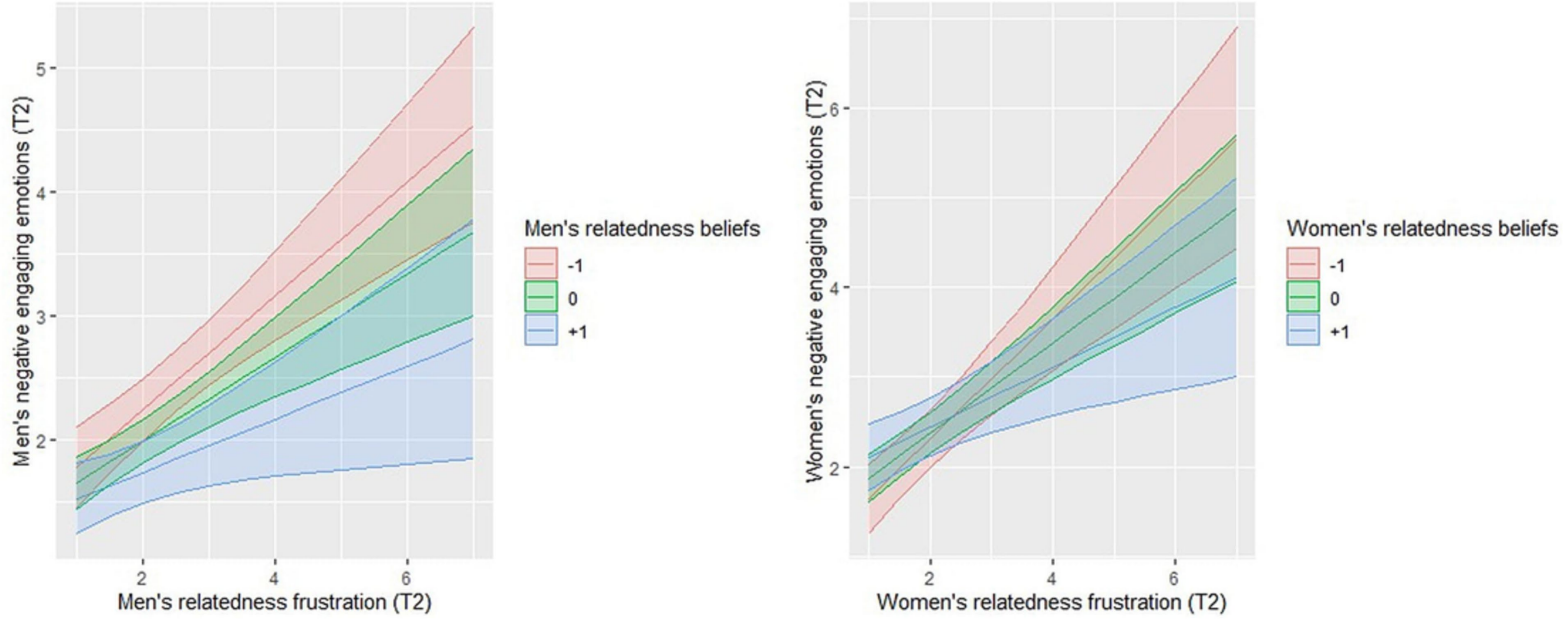
The interaction effects of relatedness beliefs with relatedness frustration on negative engaging emotions for both genders. Note: Lines represent the simple slopes of low (−1 SD) and high (+1 SD) relatedness beliefs. Analyses were conducted with unstandardized coefficients. The colors represent the 95% confidence intervals.

Simple slopes analyses revealed that the positive association between relatedness frustration and negative engaging emotions at low levels of relatedness beliefs was positive and significant for men (*B* = 2.93, SE = 1.24, *p* < 0.05) and women (*B* = 0.96, SE = 0.29, *p* < 0.01). For high levels of relatedness beliefs, the associations between relatedness frustration and negative engaging emotions was positive and significant for men (*B* = 1.27, SE = 0.23, *p* < 0.001) but not for women (*B* = 0.78, SE = 0.40, *p* = 0.060). These analyses implied that individuals with high relatedness beliefs, reported less negative engaging emotions when experiencing higher levels of relatedness frustration than people who were high in frustration, and that attributed less importance to this relational need (Figure 4). Relatedness relationship beliefs were not a significant moderator of the partner effects for men or women.

## 4 Discussion

The current findings provide initial support for our hypothesis that partners’ emotional experiences during conflict can be – at least in part – understood from the frustration of some of their core relational needs. More specifically, we found that both men and women experienced more negative disengaging emotions – anger and irritation – when their autonomy needs were frustrated during conflict. Similarly, men and women experienced more negative engaging emotions – sadness, hurt, disappointment – when their relatedness needs were frustrated during conflict. These findings were in line with our predictions and suggest that the kind of negative emotions partners experience during relationship conflict is associated with the specific relational need that is frustrated, suggesting that different emotions may indeed serve as alarms when specific relational needs are unmet.

Although we found evidence for the association between partners’ level of autonomy/relatedness frustration and the concurrent experience of negative (dis)engaging emotions during conflict, we did not find evidence that these emotions predicted a decrease in relational frustration over the course of the conflict. We hypothesized that partners’ experience and expression of disengaging emotions towards one another, potentially would foster social distancing behaviors and restore self-independence, thereby reducing autonomy frustration. By the same token, we expected that engaging emotions, would foster mutual cooperative behavior, promoting and/or restoring a sense of closeness and harmony, thereby reducing relatedness frustration. On the contrary, we found – at least for men – that higher levels of negative engaging emotions, as reported at the beginning of the conflict, were predictive of more relatedness frustration near the end of the interaction.

Three possible explanations for these finding arise. First, in our study we assessed partners’ experienced emotions, not the expressed ones. It is possible that the emotions experienced by male participants were not the same as those expressed and therefore perceived by the partners. In Western cultures, men often adhere to traditional masculine ideals that discourage the open expression of emotions (Fischer and Manstead, 2000; Fischer et al., 2004). Speculatively, men might be more likely to suppress or downplay their emotional experiences during conflict interactions, leading to a discrepancy between their internal emotional state and what is outwardly expressed. This mismatch could have influenced the communication regarding the frustration of one’s relational needs to the partner who consequently did not enact behaviors to meet them, thereby increasing their frustration. Second, negative (dis)engaging emotions might only be predictive of a decrease in frustration during conflict when these needs are frustrated to a significant degree or for a significant period of time. In our sample, the level of interaction-based need frustration was rather low and the research design focused on a limited time window. The short time frame may have limited the opportunity for participants to receive feedback and to engage in iterative processes. Effective emotion and frustration regulation involve continuous monitoring and adjustment, based on feedback from one’s own emotions and the reactions of the partner (Yuan et al., 2015; Naragon-Gainey et al., 2017). With only a short period of time that was assessed, participants may not have had sufficient feedback from their partner or time to adapt their regulation strategies and responses, which hindered us to capture the complete unfolding of the regulatory process. Third, it is also possible that individual differences, such as attachment style, might impact how partners emotionally react when their or their partner’s relational needs are frustrated during conflict. Previous studies showed how partners with anxious attachment styles, characterized by heightened emotional sensitivity and a strong desire for closeness, may be more vulnerable to experiencing emotional distress when their relational needs are unmet (Benson et al., 2013; Gökdağ, 2021). This heightened emotional reactivity, in turn, could contribute to elevated need frustration as they experience intensified negative emotions when their relational needs go unmet (Imran and Jackson, 2022). In contrast, individuals with avoidant attachment styles, who prioritize emotional self-sufficiency and independence, may exhibit emotional distancing when their needs go unmet (Kirby et al., 2005; Domingue and Mollen, 2009). This emotional distancing could intensify their sense of autonomy need frustration, as their emotional self-sufficiency may be hindered by the perceived emotional demands of their partner. Future studies should take into consideration the role of attachment styles in shaping the emotions-frustration association to provide a more nuanced understanding of the emotion dynamics within romantic relationships.

We found that a person’s relatedness beliefs play a role in the experience of negative engaging emotions due to relatedness frustration during conflict. For people who considered relatedness to be important, negative engaging emotions were not so strongly associated with relatedness frustration as for people low on relatedness beliefs. This was not in line with our prediction, but might result from the fact that individuals who place more importance on relatedness within their relationship, might cope better – and more constructively – with their relatedness frustration, enacting self-regulatory mechanisms that do not elicit such a strong emotional experience of negative engaging emotions, and prevent distancing (Rusbult et al., 1991; Harper and Welsh, 2007; Buck and Neff, 2012). Moreover, it is possible that individual differences such as heightened awareness and attunement to relational dynamics, and adaptive and constructive interpersonal skills, might also moderate gender differences found. Investigating these individual differences in future studies could shed light on whether men who hold more beliefs valuing relatedness may exhibit enhanced abilities in utilizing emotion as information, expressing their emotions, and engaging in self and co-regulation processes.

Finally, the absence of significant partner effects in the frustration-emotion association might be explained by the fact that rather than the actual values of frustration and specific emotions as auto-reported by partners, an individual’s “perception” of partners’ needs frustration and emotions matters. Partners may have experienced specific levels of relational frustration and emotions, but not expressed them, making it hard for the other partner to perceive them. For example, if an individual perceived that the partner was experiencing a low level of relational need frustration or specific emotions, this perception may have influenced how the individual responded emotionally during the conflict situation, even if the partner did report high levels of need frustration or that specific emotional experience themselves. Taken together, our findings suggest that the association between emotions and need frustration is – at least in the short term – mainly determined by one’s own experiences during conflict.

### 4.1 Limitations and future research

Being the first study that directly investigates the association between partners’ interaction-based need frustration and the experience of (dis)engaging emotions in partners, several limitations should be considered. First, due to the set-up of the study, interaction-based need frustration and emotional experience were assessed only twice during the video-review task, and within a time-interval of 5 min. It is possible that relevant degrees of need frustration and emotions occurred that were not captured at these two points (T_2_ and T_5_). It would be therefore valuable to replicate these findings using continuous measures assessing longer time spans in order to better capture the interaction’s dynamics. Such measurements would also allow to more properly investigate the temporal characteristics of the frustration-emotion association. Second, the present study was set in a laboratory environment in which couples discussed negative topics regarding their couple relationship and thereafter performed a video-mediated recall task. However, this paradigm has been shown in previous research to often elicit limited emotional responses in participants (Ickes et al., 2000; Gordon and Chen, 2014). Future studies with different methods, such as experience sampling methods, are needed to generalize our findings across different types of interpersonal situations and naturally occurring interactions. Third, while a range of conflict topics common in romantic relationships was examined, the current study did not pre-test for nor differentiated between these conflict topics based on partners’ perceived severity of the conflict. Consequently, the potential influence of conflict severity on partners’ needs frustration, and emotional responses remains unexplored. Future observational research should aim to deal with this limitation. Finally, our study was based on a convenience sample of western, middle-class, and heterosexual couples, thereby limiting the generalizability of the results. Consequently, future research is needed to replicate these findings with more heterogeneous samples and with cross-cultural validation, especially in cultures varying in the importance of in (ter) dependence relational needs and (dis)engaging emotions (Mesquita et al., 2017; Schouten et al., 2020).

## 5 Conclusion

The present study provides first direct evidence that partners’ emotional experience varies according to the frustration of their own relational needs during conflict. While autonomy frustration in partners concurred with the experience of more negative disengaging emotions such as anger and irritation, relatedness frustration went together with experiencing more negative engaging emotions such as hurt, sadness, and disappointment. The importance that partners attribute to relatedness within relationships in general, influenced the experience of negative engaging emotions resulting from the frustration of this particular need, whereas this did not apply to the importance that partners attribute to autonomy with regard to the association between autonomy frustration and the experience of negative disengaging emotions. Furthermore, the experience of negative disengaging emotions did not influence the frustration of the need for autonomy during the conflict, while the experience of negative engaging emotions positively predicted relatedness frustration during the interaction, but only for men. Although future research should uncover further nuances, our findings provide promising insight into how emotional experience may vary as a function of intimate relationship needs. This knowledge can increase the awareness of couple therapists in adopting a needs perspective during the case-formulation and intervention stages of therapy as it may allow them to focus on more covert underlying relational issues.

## Data availability statement

The datasets presented in this study can be found in online repositories. The names of the repository/repositories and accession number (s) can be found at: osf.io/cuvj8.

## Ethics statement

The studies involving humans were approved by Ethical Committee of the Faculty of Psychology and Educational Sciences of Ghent University. The studies were conducted in accordance with the local legislation and institutional requirements. The participants provided their written informed consent to participate in this study.

## Author contributions

DP: conceptualization, validation, formal analysis, data curation, writing – original draft, and visualization. LS: conceptualization, methodology, validation, formal analysis, review and editing, and supervision. LV: resources, review and editing, supervision, project administration, and funding acquisition. All authors contributed to the article and approved the submitted version.

## Footnotes

^1^Relational goals and needs are interconnected and complementary concepts within the field of psychology and interpersonal relationships. Goals represent the desired outcomes or objectives individuals aim to achieve in their relationships, encompassing emotional, cognitive, and behavioral aspects, whereas needs refer to the fundamental psychological requirements individuals seek to fulfill within their relationships for their well-being and satisfaction (Collins et al., 2006; Denzinger et al., 2018). Since our study relies on SDT, throughout this manuscript the term need is used to analyze and explore the multifaceted nature of human relationships, incorporating aspirations and fundamental psychological requirements.

^2^According to SDT, people also have the need to feel competent and effective at what they do (Deci and Ryan, 2014). However, competence appears to be a less central predictor in intimate relationships (Patrick et al., 2007; Vanhee et al., 2018). People often have ways to feel competent that are not within their intimate relationships, such as in work, school, or leisure (La Guardia et al., 2000). Furthermore, the need for autonomy and relatedness better capture the two poles of the social engagement continuum of emotion, thus allowing us to have a clear theoretical argument for our prediction. Given the specific focus of our research on emotional experiences within intimate relationships, focusing on autonomy and relatedness enables a more comprehensive understanding of the emotional dynamics in this context. For these reasons, we did not include a measure of competence in our research (interested readers can find the results of these analyses in the Supplementary Tables S3–S6).

^3^The Bayesian Information Criterion (BIC) and the Akaike Information Criterion (AIC) are goodness-of-fit measures that are corrected for model complexity (Field, 2009). Models with smaller BIC and AIC values provide a better fit-complexity balance.
