# Supplementary material for: Relational needs frustration: an observational study on the role of negative (dis)engaging emotions
Source: Front Psychol. 2023 Nov 23;14:1232125. doi: 10.3389/fpsyg.2023.1232125 (PMC10701550; doi:10.3389/fpsyg.2023.1232125)
Supplement: Supplementary file 1 [file Data_Sheet_1.PDF]

Table 1S. Results for the APIM predicting autonomy frustration (T_5) from men's and women's negative disengaging emotions (T_2), controlling for autonomy frustration and negative engaging emotions at previous time during the interaction (T_2).

Model 2a parameters	Estimate	SE	95% CI
Intercepts			
Men	2.34***	0.12	[2.11; 2.57]
Women	2.16***	0.11	[1.93; 2.38]
Actor effects			
Disengaging emotions$_{mT2}$ → Autonomy frustration$_{mT5}$	0.02	0.12	[–0.22; 0.25]
Disengaging emotions$_{wT2}$ → Autonomy frustration$_{wT5}$	–0.11	0.09	[–0.29; 0.08]
Engaging emotions$_{mT2}$ → Autonomy frustration$_{mT5}$	0.02	0.12	[–0.22; 0.26]
Engaging emotions$_{wT2}$ → Autonomy frustration$_{wT5}$	0.32***	0.10	[0.12; 0.53]
Autonomy frustration$_{mT2}$ → Autonomy frustration$_{mT5}$	0.44***	0.09	[0.27; 0.61]
Autonomy frustration$_{wT2}$ → Autonomy frustration$_{wT5}$	0.44***	0.08	[0.28; 0.61]
Partner effects			
Disengaging emotions$_{mT2}$ → Autonomy frustration$_{wT5}$	–0.18	0.10	[–0.37; 0.01]
Disengaging emotions$_{wT2}$ → Autonomy frustration$_{mT5}$	0.03	0.11	[–0.20; 0.27]
Engaging emotions$_{mT2}$ → Autonomy frustration$_{wT5}$	0.11	0.10	[–0.10; 0.31]
Engaging emotions$_{wT2}$ → Autonomy frustration$_{mT5}$	–0.10	0.12	[–0.33; 0.14]
Autonomy frustration$_{mT2}$ → Autonomy frustration$_{wT5}$	0.18*	0.09	[0.01; 0.34]
Autonomy frustration$_{wT2}$ → Autonomy frustration$_{mT5}$	0.06	0.08	[–0.11; 0.22]

Note: $^{*}p < .05$; $^{**}p < .01$; $^{***}p < .001$

Table 2S. Results for the APIM predicting relatedness frustration (T_5) from men's and women's negative engaging emotions (T_2), controlling for relatedness frustration and negative disengaging emotions at previous time during the interaction (T_2).

Model 2b parameters	Estimate	SE	95% CI
Intercepts			
Men	2.05***	0.09	[1.87; 2.23]
Women	2.00***	0.10	[1.81; 2.18]
Actor effects			
Engaging emotions$_{mT2}$ → Relatedness frustration$_{mT5}$	0.36***	0.11	[0.15; 0.58]
Engaging emotions$_{wT2}$ → Relatedness frustration$_{wT5}$	0.27***	0.09	[0.09; 0.45]
Disengaging emotions$_{mT2}$ → Relatedness frustration$_{mT5}$	–0.08	0.10	[–0.28; 0.11]
Disengaging emotions$_{wT2}$ → Relatedness frustration$_{wT5}$	–0.21*	0.08	[–0.37; 0.05]
Relatedness frustration$_{mT2}$ → Relatedness frustration$_{mT5}$	0.54***	0.07	[0.39; 0.68]
Relatedness frustration$_{wT2}$ → Relatedness frustration$_{wT5}$	0.75***	0.07	[0. 06; 0.89]
Partner effects			
Engaging emotions$_{mT2}$ → Relatedness frustration$_{wT5}$	–0.07	0.09	[–0.24; 0.11]
Engaging emotions$_{wT2}$ → Relatedness frustration$_{mT5}$	0.04	0.11	[–0.17; 0.26]
Disengaging emotions$_{mT2}$ → Relatedness frustration$_{wT5}$	0.02	0.08	[–0.14; 0.18]
Disengaging emotions$_{wT2}$ → Relatedness frustration$_{mT5}$	–0.20*	0.10	[–0.41; 0.01]
Relatedness frustration$_{mT2}$ → Relatedness frustration$_{wT5}$	–0.06	0.07	[–0.21; 0.08]
Relatedness frustration$_{wT2}$ → Relatedness frustration$_{mT5}$	0.06	0.08	[–0.10; 0.21]

Note: $^{*}p < .05$; $^{**}p < .01$; $^{***}p < .001$

Table 3S. Results for the APIM predicting negative disengaging emotions (T_2) from men's and women's competence frustration (T_2), controlling for global competence frustration.

**Model 1c parameters**	**Estimate**	**SE**	**95% CI**
Intercepts			
Men	1.93^{***}	0.09	[1.75; 2.12]
Women	2.22^{***}	0.11	[2.00; 2.44]
Actor effects			
Competence frustration$_{mT2}$ → Disengaging emotions$_{mT2}$	0.24^{***}	0.06	[0.11; 0.36]
Competence frustration$_{wT2}$ → Disengaging emotions$_{wT2}$	0.42^{***}	0.09	[0.25; 0.59]
Global competence frustration$_{m}$ → Disengaging emotions$_{mT2}$	0.35^{**}	0.14	[0.08; 0.62]
Global competence frustration$_{w}$ → Disengaging emotions$_{wT2}$	0.35^{*}	0.16	[0.04; 0.66]
Partner effects			
Competence frustration$_{mT2}$ → Disengaging emotions$_{wT2}$	0.12	0.07	[–0.02; 0.26]
Competence frustration$_{wT2}$ → Disengaging emotions$_{mT2}$	0.08	0.08	[–0.07; 0.23]
Global competence frustration$_{m}$ → Disengaging emotions$_{wT2}$	0.08	0.13	[–0.17; 0.34
Global competence frustration$_{w}$ → Disengaging emotions$_{mT2}$	0.49	0.16	[0.17; 0.82]

Note: $^{*}p < .05$; $^{**}p < .01$; $^{***}p < .001$

Table 4S. Results for the APIM predicting negative engaging emotions (T_2) from men's and women's competence frustration (T_2), controlling for global competence frustration.

**Model 1d parameters**	**Estimate**	**SE**	**95% CI**
Intercepts			
Men	1.98^{***}	0.09	[1.80; 2.15]
Women	2.36^{***}	0.11	[2.13; 2.58]
Actor effects			
Competence frustration$_{mT2}$ → Engaging emotions$_{mT2}$	0.31^{***}	0.06	[0.19; 0.42]
Competence frustration$_{wT2}$ → Engaging emotions$_{wT2}$	0.48^{***}	0.09	[0.31; 0.65]
Global competence frustration$_{m}$ → Engaging emotions$_{mT2}$	0.33^{*}	0.12	[0.08; 0.59]
Global competence frustration$_{w}$ → Engaging emotions$_{wT2}$	0.27	0.16	[–0.05; 0.58]
Partner effects			
Competence frustration$_{mT2}$ → Engaging emotions$_{wT2}$	0.02	0.07	[–0.12; 0.15]
Competence frustration$_{wT2}$ → Engaging emotions$_{mT2}$	0.05	0.08	[–0.10; 0.20]
Global competence frustration$_{m}$ → Engaging emotions$_{wT2}$	0.23	0.12	[–0.02; 0.47]
Global competence frustration$_{w}$ → Engaging emotions$_{mT2}$	0.39^{*}	0.17	[0.06; 0.71]

Note: $^{*}p < .05$; $^{**}p < .01$; $^{***}p < .001$

Table 5S. Results for the APIM predicting competence frustration (T_5) from men's and women's negative disengaging emotions (T_2), controlling for competence frustration at previous time during the interaction (T_2).

Model 2c parameters	Estimate	SE	95% CI
Intercepts			
Men	1.85***	0.09	[1.67; 2.02]
Women	1.77***	0.09	[1.60; 1.95]
Actor effects			
Disengaging emotions$_{mT2}$ → Competence frustration$_{mT5}$	0.17*	0.08	[0.02; 0.33]
Disengaging emotions$_{wT2}$ → Competence frustration$_{wT5}$	–0.21	0.06	[–0.15; 0.10]
Competence frustration$_{mT2}$ → Competence frustration$_{mT5}$	0.48***	0.06	[0.36; 0.60]
Competence frustration$_{wT2}$ → Competence frustration$_{wT2}$	0.72***	0.07	[0.59; 0.86]
Partner effects			
Disengaging emotions$_{mT2}$ → Competence frustration$_{wT5}$	0.04	0.06	[–0.09; 0.16]
Disengaging emotions$_{wT2}$ → Competence frustration$_{mT5}$	–0.16*	0.08	[–0.32; –0.01]
Competence frustration$_{mT2}$ → Competence frustration$_{wT5}$	–0.05	0.07	[–0.19; 0.09]
Competence frustration$_{wT2}$ → Competence frustration$_{mT2}$	–0.02	0.06	[–0.14; 0.10]

Note: $^{*}p < .05$; $^{**}p < .01$; $^{***}p < .001$

Table 6S. Results for the APIM predicting competence frustration (T_5) from men's and women's negative engaging emotions (T_2), controlling for competence frustration at previous time during the interaction (T_2).

Model 2d parameters	Estimate	SE	95% CI
Intercepts			
Men	1.86^{***}	0.09	[1.69; 2.04]
Women	1.78^{***}	0.09	[1.61; 1.96]
Actor effects			
Engaging emotions$_{mT2}$ → Competence frustration$_{mT5}$	0.22^{**}	0.09	[0.05; 0.39]
Engaging emotions$_{wT2}$ → Competence frustration$_{wT5}$	–0.01	0.07	[–0.15; 0.13]
Competence frustration$_{mT2}$ → Competence frustration$_{mT5}$	0.45^{***}	0.06	[0.33; 0.57]
Competence frustration$_{wT2}$ → Competence frustration$_{wT2}$	0.70^{***}	0.07	[0.56; 0.85]
Partner effects			
Engaging emotions$_{mT2}$ → Competence frustration$_{wT5}$	0.05	0.07	[–0.08; 0.19]
Engaging emotions$_{wT2}$ → Competence frustration$_{mT5}$	–0.05	0.09	[–0.23; 0.12]
Competence frustration$_{mT2}$ → Competence frustration$_{wT5}$	–0.04	0.07	[–0.18; 0.10]
Competence frustration$_{wT2}$ → Competence frustration$_{mT2}$	–0.04	0.06	[–0.17; 0.08]

Note: $^{*}p < .05$; $^{**}p < .01$; $^{***}p < .001$

Table S7. Results for the APIMs predicting negative disengaging and engaging emotions (T_5) from men's and women's autonomy and relatedness frustration (T_2), controlling for global relational need frustration.

**Model 1e parameters**	**Estimate**	**SE**	**95% CI**
Intercepts			
Men	2.00***	0.11	[1.79; 2.21]
Women	2.37***	0.12	[2.14; 2.61]
Actor effects			
Autonomy frustration$_{mT2}$ → Disengaging emotions$_{mT5}$	0.22***	0.07	[0.07; 0.37]
Autonomy frustration$_{wT2}$ → Disengaging emotions$_{wT5}$	0.24***	0.08	[0.07; 0.41]
Global autonomy frustration$_{m}$ → Disengaging emotions$_{mT5}$	0.13	0.16	[–0.18; 0.44]
Global autonomy frustration$_{w}$ → Disengaging emotions$_{wT5}$	0.59***	0.18	[0.24; 0.93]
Partner effects			
Autonomy frustration$_{mT2}$ → Disengaging emotions$_{wT5}$	0.12	0.08	[–0.03; 0.28]
Autonomy frustration$_{wT2}$ → Disengaging emotions$_{mT5}$	0.04	0.08	[–0.12; 0.20]
Global autonomy frustration$_{m}$ → Disengaging emotions$_{wT5}$	0.25	0.16	[–0.07; 0.56]
Global autonomy frustration$_{w}$ → Disengaging emotions$_{mT5}$	0.31	0.17	[–0.03; 0.65]

**Model 1f parameters**	**Estimate**	**SE**	**95% CI**
Intercepts			
Men	1.96***	0.10	[1.75; 2.16]
Women	2.40***	0.12	[2.15; 2.65]
Actor effects			
Relatedness frustration$_{mT2}$ → Engaging emotions$_{mT5}$	0.36***	0.08	[0.20; 0.51]
Relatedness frustration$_{wT2}$ → Engaging emotions$_{wT5}$	0.33***	0.09	[0.15; 0.51]
Global relatedness frustration$_{m}$ → Engaging emotions$_{mT5}$	–0.06	0.20	[–0.48; 0.34]
Global relatedness frustration$_{w}$ → Engaging emotions$_{wT5}$	0.51	0.26	[–0.01; 1.02]
Partner effects			
Relatedness frustration$_{mT2}$ → Engaging emotions$_{wT5}$	0.06	0.08	[–0.09; 0.21]
Relatedness frustration$_{wT2}$ → Engaging emotions$_{mT5}$	0.02	0.09	[–0.17; 0.20]
Global relatedness frustration$_{m}$ → Engaging emotions$_{wT5}$	0.03	0.22	[–0.40; 0.46]
Global relatedness frustration$_{w}$ → Engaging emotions$_{mT5}$	0.07	0.24	[–0.41; 0.55]

Note: $^{*}p < .05$; $^{**}p < .01$; $^{***}p < .001$
